# Subperiosteal Orbital Hematoma: Imaging Findings of a Rare Complication of Sickle Cell Disease: Subperiosteal orbital hematoma is a rare entity mainly seen in pediatric patients with sickle cell disease and occurs secondary to local vascular disturbances following facial bone infarction

**DOI:** 10.5334/jbsr.1786

**Published:** 2019-06-28

**Authors:** Nick Van de Voorde, Paul M. Parizel, Sven Dekeyzer

**Affiliations:** 1 UZA, BE

**Keywords:** subperiosteal orbital hematoma, sickle cell disease, facial bone infarction, vaso-occlusive crisis

## Abstract

Sickle cell disease is the most common hemoglobinopathy. Homozygous patients are prone to vaso-occlusive crises. A 19-year-old male patient with the homozygous sickle cell trait was admitted to the hospital due to a sickle cell crisis. During his admission he developed a left periorbital edema. The diagnosis of a subperiosteal orbital hematoma (SOH) was made by CT and MRI imaging. SOH is a rare complication of a VOC. The clinical course is mostly self-resolving, with some cases reporting the need for surgical decompression when orbital compression syndrome is clinically diagnosed. Differentiation between infection on imaging is necessary for further treatment.

## Introduction

Sickle cell disease (SCD) is a group of inherited hemolytic disorders characterized by mutations in the hemoglobin β subunit. Under conditions of deoxygenation, hemoglobin-tetramers with two of these mutant β-globin proteins tend to polymerize, causing the red bloods cells to assume a sickle or crescentic shape. SCD is inherited as an autosomal recessive trait. Individuals who are heterozygous for the β-allele carry the sickle cell trait, but do not have SCD. Individuals who are homozygous for the β-allele have sickle cell anemia. SCD is the most common hemoglobinopathy in the world and is most prevalent in sub-Saharan Africa, where up to 30% people exhibit the sickle cell trait [1]. β-allele heterozygosity generates a relative survival advantage to malaria. SCD homozygotes are prone to vaso-occlusive crises (VOC). When the crescentic red blood cells become trapped in the capillaries, infarction may occur. Lungs, spleen, kidneys and long bones or vertebrae are a common site of infarction. Facial bones, on the other hand, are infrequently involved due to their narrow marrow spaces. We present a case of an uncommon complication of a VOC in a patient with SCA.

## Case Presentation

**Figure 1:**
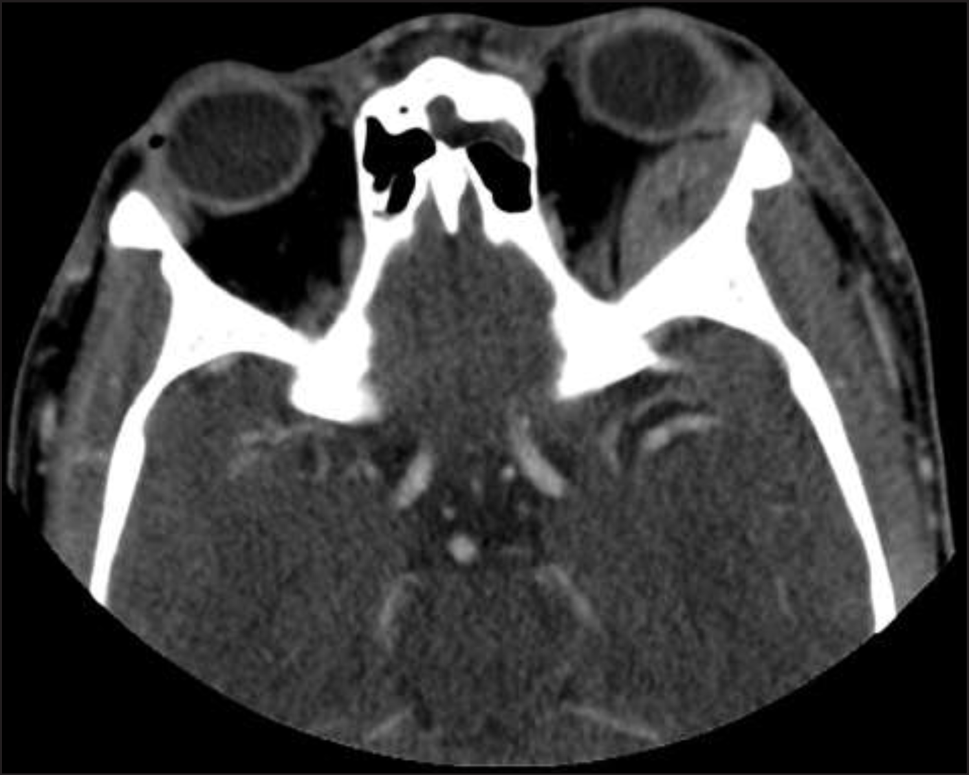
Axial contrast-enhanced CT: A biconcave lesion lining the superolateral left orbital wall.

**Figure 2:**
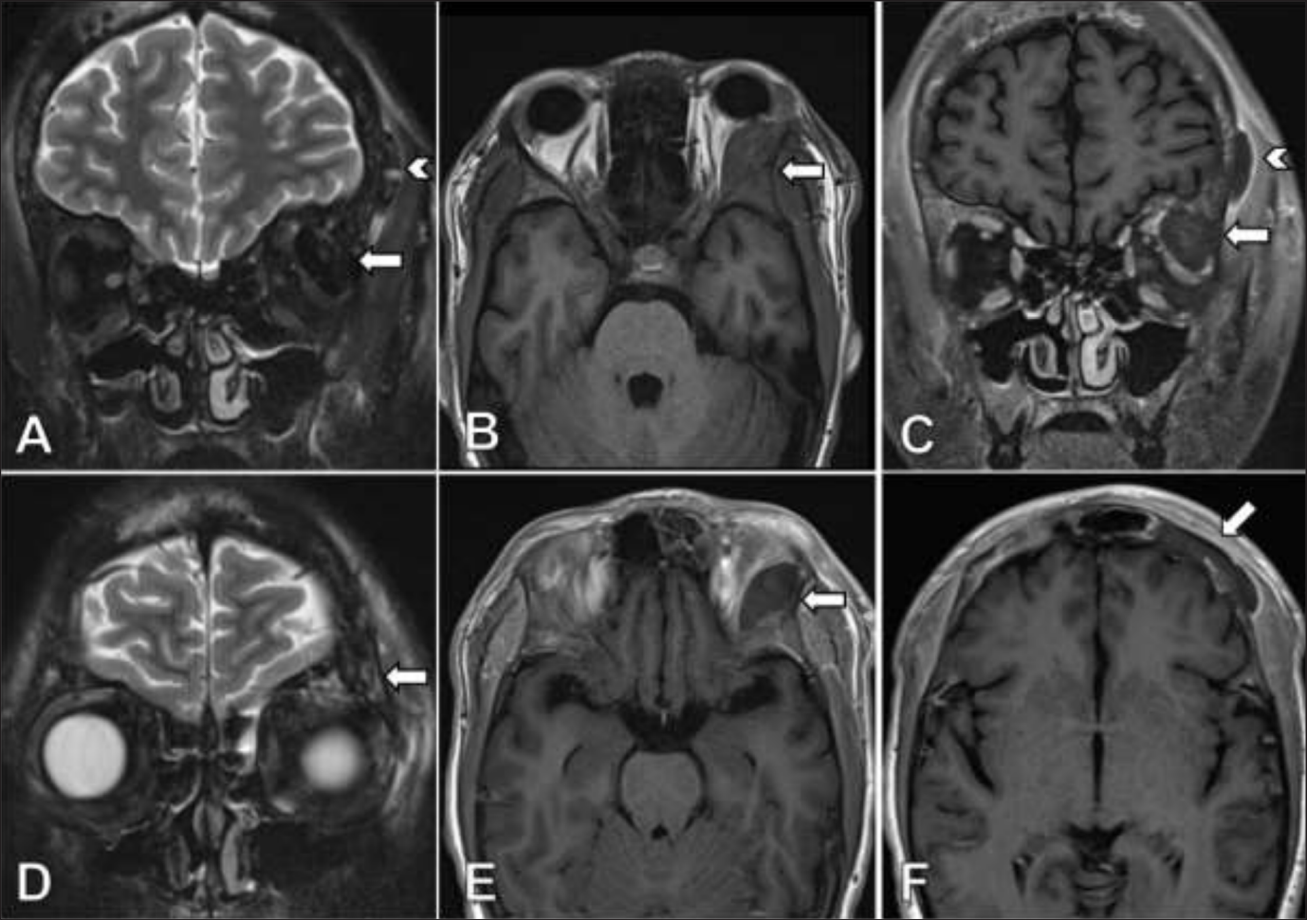
Coronal TSE T2-weighted image with fat suppression **(A)** shows a markedly hypointense lesion located in the lateral aspect of the left orbital roof (arrow) and a second lesion lining the external table of the frontal bone on the left side (arrow head). On axial TSE T1-weighted images **(B)** the lesion exhibits a isointense signal compared to the adjacent bone, without lesional enhancement after injection of gadolinium **(E)**. Coronal contrast-enhanced TSE T1-weighted image **(C)** reveals a faint perilesional enhancement, more prominently in the lesion lining the frontal bone on the left (arrow head). Coronal TSE T2-weighted image with fat suppression **(D)** shows asymmetrical, discrete bone edema and prominent nonenhancing lesions on contrast-enhanced TSE T1-weighted image **(F)** in the frontal bone on the left side compared to the right side.

A 19-year-old male patient with the homozygous sickle cell trait was admitted to the hospital due to a sickle cell crisis. His main complaint was unbearable pain in the extremities. The patient’s history was remarkable for multiple previous admissions for sickle cell crises. During his admission he developed a swollen left eye, with discrete ptosis of the upper eyelid and minimal exophthalmia. There were no visual disturbances and eye movement was unimpaired. A contrast-enhanced computed tomography (CT) of the orbits showed a lens shaped extraconal mass lining the lateral wall of the left orbit (Figure 1). The lesion measured 3.1 × 1.2 cm with high attenuation due to enhancement or spontaneously dense compounds. The underlying frontal and sphenoid bones were unremarkable. The patient was referred for MRI the same day for further work-up. The lesion was markedly hypointense on T2-weighted images with fat suppression (Figure 2A). T1-weighted sequences showed an isointense signal comparable to the adjacent bone (Figure 2B). There was no lesional enhancement after injection of gadolinium and faint perilesional enhancement (Figure 2C). Imaging findings were compatible with an acute subperiosteal orbital hematoma (SOH). Additionally, MRI revealed a new extracranial subperiosteal hematoma lining the external table of the frontal bone on the left side (Figure 2C). This hematoma was less hypointense on T2-weighted images and exhibited more prominent perilesional enhancement. The frontal bone and left greater wing of the sphenoid bone showed discrete bone oedema on T2-weighted images (Figure 2D) and asymmetrical low signal intensity on contrast-enhanced T1-weighted images (Figure 2F), suggesting areas of infarction. The patient received supportive treatment after diagnosis, and the eye swelling diminished spontaneously over time.

## Discussion

Facial bone infarction is rare in SCD patients and SOH is even more infrequent. Both are mainly seen in pediatric patients, who have a greater marrow space compared to older individuals. Bilateral orbital involvement is reported in 42% of cases [2]. Bone infarction complicated by a hematoma are believed to be a unique feature of facial bone infarction, with the hematoma located either in the periost extracranially or in the epidural space intracranially. The etiology of this paradoxical hemorrhage in infarcted bone is unclear. A rupture of vessel walls due to necrosis has been suggested [3]. However, a pressure build-up due to obstructed vessels transmitted to the periost with subsequent bursting of the bridging veins is more likely [2]. Non-traumatic subperiosteal hematoma is also known to occur in patients with bleeding disorders and cases of sudden increased orbital venous pressure, such as strangulation, delivery, vomiting, and barotrauma in divers [2]. The SOH is almost always located in the orbital roof, which may be due to loose periosteal attachment site or the anatomical location of venous foramina known as ‘cribra orbitalia’, present in 11–25% of studied skulls [4].

Patients with SCD are relatively immunocompromised and therefore more susceptible to infections. Orbital imaging and correct interpretation in SCD patients presenting with orbital swelling is paramount for differentiation between hematoma and infection. A SOH can be managed with supportive corticosteroid therapy, which is contraindicated with infection. Surgical decompression is indicated when SOH is associated by impaired ocular movement and deteriorating vision, termed orbital compression syndrome. Infection needs to be tackled with aggressive antibiotic treatment, and abscess may require drainage.

Imaging is important for the differential. Our patient received prompt CT and MRI evaluation on the same day. Although the lesion was hyperdense on CT, we could not be sure if this represented blood or contrast enhancement as only contrast-enhanced CT was performed. The signal characteristics on MRI of this nonenhancing lesion combined with the acute clinical presentation were compatible with an acute hematoma, hence the diagnosis of a subperiosteal hematoma. The markedly T2-hypointense signal made a neoplastic lesion less likely.

The main differential diagnosis is infection. Orbital cellulitis will show diffuse infiltration of orbital and/or periorbital tissue with enhancement. Periosteal abscesses will present as a hypodense biconcave mass with peripheral enhancement, in which gas inclusions can be present. Periosteal abscesses are often associated with sinusitis, which was not present in our patient. Differentiation between facial bone infarction and osteomyelitis is a diagnostic challenge. Both can present with bone oedema, soft tissue enhancement, and collections. When cortical defects are present diagnosis of osteomyelitis is more likely.

## Conclusion

In our case report a patient with known SCD was admitted to the hospital for a SCC, during which he developed left periorbital oedema. This was caused by a subperiosteal orbital hematoma secondary to infarction of the facial bones. Imaging findings of a SOH include a (spontaneously) hyperdense biconcave lesion lining the superior orbital roof on CT and T1- and T2-weighted signal intensities of hemorrhage related to the time of onset. The underlying bone shows heterogeneous signal intensities due to infarction. Differentiation between infection and hematoma is crucial. The patient was treated conservatively and the periorbital oedema regressed. No control imaging was performed.

## References

[1] Ware, RE, de Montalembert, M, Tshilolo, L and Abboud, MR. Sickle cell disease. Lancet [Internet]. 2017 7 15; 390(10091): 311–23. Available from: http://www.ncbi.nlm.nih.gov/pubmed/28159390 Accessed 2019 Feb 10. DOI: 10.1016/S0140-6736(17)30193-9 28159390

[2] McNab, AA. Nontraumatic orbital hemorrhage. Surv Ophthalmol [Internet]. 2014 3; 59(2): 166–84. Available from: http://www.ncbi.nlm.nih.gov/pubmed/24359805 Accessed 2019 Feb 2. DOI: 10.1016/j.survophthal.2013.07.002 24359805

[3] Karacostas, D, Artemis, N, Papadopoulou, M and Christakis, J. Case report: Epidural and bilateral retroorbital hematomas complicating sickle cell anemia. Am J Med Sci [Internet]. 1991 8; 302(2): 107–9. Available from: http://www.ncbi.nlm.nih.gov/pubmed/1897555 Accessed 2019 Jan 23. DOI: 10.1097/00000441-199108000-00008 1897555

[4] Whittnall, SE. Part I. Osteology. The bones forming the orbit, its relations, and the accessory air-sinuses of the nose In: Whittnall, SE (ed.), The Anatomy of the Human Orbit and Accessory Organs of Vision. London, Oxford University Press; ed 2, 1932: 32e3.

